# Supplementary material for: Influenza and Other Prophylactic Vaccination Coverage in Polish Adult Patients Undergoing Allergen Immunotherapy—A Survey Study among Patients and Physicians
Source: Vaccines (Basel). 2022 Apr 8;10(4):576. doi: 10.3390/vaccines10040576 (PMC9027432; doi:10.3390/vaccines10040576)
Supplement: Supplementary file 1 [file vaccines-10-00576-s001.zip › File S1.pdf]

Vaccinations against infectious diseases in patients undergoing allergen immunotherapy

1. Please select your age range:
   - 18-29
   - 30-39
   - 40-49
   - 50-59
   - 60-69
   - 70-79
   - more than 80 years old

2. What are you allergic to?
   - respiratory allergens
   - food
   - drugs
   - insect venom
   - other allergy

3. How long have you been undergoing allergen immunotherapy?
- 6 months – 1 year
   - 1-2 years
   - 2-3 years
   - more than 3 years

4. What type of allergen immunotherapy are you undergoing?
- sublingual
   - subcutaneous
   - sublingual and subcutaneous

5. Have you ever experienced any allergic adverse reactions because of allergen immunotherapy?
- urticaria
   - allergic rhinitis/ conjunctivitis
   - dyspnea
   - anaphylactic shock
   - no, I haven't

6. Do you suffer from any of the following chronic diseases?
- asthma
   - hypertension
   - heart failure
   - diabetes
   - cancer
   - renal failure

7. Are you satisfied with the effectiveness of allergen immunotherapy (scale 0-5, where: 0 - I am not satisfied, 5 - I am very satisfied)
- 0
   - 1
   - 2
   - 3
   - 4
   - 5

8. Do you live with children under 14 years old?

- yes
- no

9. Do you consider yourself as a person more vulnerable to influenza/ influenza-like illnesses compared to the people from your closest surroundings?
- yes
- no

10. Does the fact that you undergo allergen immunotherapy have any impact on your treatment of other diseases?
- it makes it more difficult
- there is no impact

11. Have you ever got vaccinated against influenza? If yes - then how many times?
- no, I haven't
- once
- two times
- more than two times

12. Have you got vaccinated against influenza since the beginning of allergen immunotherapy?
- yes
- no

13. Did you get vaccinated against influenza during the 2019/2020 season?
- yes
- no

14. Did you get vaccinated against influenza during the 2020/2021 season?
- yes
- no

15. Have you ever experienced any allergic adverse reactions because of the influenza vaccine?
- urticaria
- allergic rhinitis/ conjunctivitis
- dyspnea
- anaphylactic shock
- no, I haven't experienced any allergic adverse events after influenza vaccination

16. Have you ever experienced any local adverse reactions because of the influenza vaccine?
- pain
- swelling
- reddness

at the injection site.

17. Did these symptoms have any impact on your activity?
- they did not have any impact
- they limited my activity a little
- they significantly reduced my activity

18. Have you ever experienced any systemic adverse reactions because of the influenza vaccine?
- myalgia

- arthralgia
- fever/ subfebrile temperature
- headache
- general weakness
- other symptoms

19. Did these symptoms have any impact on your activity?
- they did not have any impact
- they limited my activity a little
- they significantly reduced my activity

20. Did you get vaccinated against tetanus in adulthood?
- yes, it was a planned vaccination
- yes, it was a vaccination after an injury
- no

21. Did you get vaccinated against *Streptococcus pneumoniae* in adulthood?
- yes
- no

22. Did you get vaccinated against pertussis <whooping cough> in adulthood (a diphtheria, pertussis, tetanus vaccine)?
- yes
- no

23. Did you get vaccinated against tick-borne encephalitis in adulthood?
- yes
- no

24. Are you willing to get vaccinated against COVID-19?
- yes
- no

25. Has it ever occurred that allergen immunotherapy collided with the performance of planned/ sudden vaccination (resulting from exposition to infectious disease)?
- yes
- no
