# Supplementary material for: Influenza and Other Prophylactic Vaccination Coverage in Polish Adult Patients Undergoing Allergen Immunotherapy—A Survey Study among Patients and Physicians
Source: Vaccines (Basel). 2022 Apr 8;10(4):576. doi: 10.3390/vaccines10040576 (PMC9027432; doi:10.3390/vaccines10040576)
Supplement: Supplementary file 1 [file vaccines-10-00576-s001.zip › File S2.pdf]

# Vaccines against infectious diseases and allergen immunotherapy

This questionnaire aims to assess the attitude of doctors who take care of patients undergoing allergen immunotherapy, towards combining allergen immunotherapy and vaccination. The questionnaire is anonymous, and takes approx. 3-5 minutes.
*Required

1/ **How often do you take care of patients undergoing allergen immunotherapy (AIT)?***
· once a week or more often
· once a week - once a month
· less often than once a month
· I haven't treated any patients undergoing AIT

2/ **In which form do your patients receive AIT?***
· subcutaneous
· sublingual
· I take care of patients receiving both forms of AIT

3/ **Does the fact that a patient undergoes AIT have any impact on their treatment because of different diseases?**
· it depends on the stage of AIT
· not all the patients admit being on AIT
· a new medication in patient's therapy makes cooperation and adherence to doctor's indications more difficult
· it doesn't have any impact
· different answer …….

4/ **Does receiving AIT have any influence on the decision about vaccinations against infectious diseases in a particular patient? ***
· it favours intentional planning of vaccinations' dates
· the planning of vaccinations' dates is more difficult
· it doesn't have any impact

5/ **While performing vaccinations against infectious diseases in a patient undergoing AIT I follow the rule: ***
· one week interval between AIT and the vaccine (AIT - one week - prophylactic vaccine OR prophylactic vaccine - one week - AIT)
· I use the Summary of Product Characteristics of the allergen extract administered during allergen immunotherapy
· I use the Summary of Product Characteristics of the vaccine
· I apply the interval according to the suggestions of patient's allergologist
· I perform vaccinations against infectious diseases regardless of the interval between AIT
· I postpone vaccinations against infectious diseases in patients undergoing AIT until the end of AIT

6/ **Do you consider vaccinations against infectious diseases safe in patients undergoing AIT?***
· yes, I know studies confirming safety of such procedures
· yes, I know recommendations of societies of allergology allowing performing vaccinations in patients undergoing AIT
· no, I think that the risk of adverse events is too high in this group of patients

7/ **Which sentence regarding the effectiveness of vaccinations against infectious diseases in patients undergoing AIT do you identify with? ***
· vaccinations are effective
· vaccinations have limited effectiveness
· vaccinations are not effective
· I have never considered this topic before

8/ **Do you recommend an annual vaccination against influenza to your patients undergoing AIT? ***
· only to the patients undergoing subcutaneous AIT
· only to the patients undergoing sublingual AIT
· to all the patients undergoing AIT regardless of the route of administration of AIT
· only to the patients with the risk of poor outcome of influenza, regardless of the route of administration of AIT
· I don't recommend vaccinations against influenza

9/ **Do you think that creating clear recommendations regarding performance of vaccinations against infectious diseases in patients undergoing AIT would be favorable in everyday doctors' practice? ***
· Yes, such recommendations would simplify performing vaccinations in patients undergoing AIT
· No, current recommendations are sufficient

10/ **I perform vaccinations against infectious diseases in:***
children adults children and adults

11/ **Please select your age range: ***
<29 years old 30-39 years old 40-49 years old >50 years old

12/ **Please select your education: ***

| | | |
|---|---|---|
| resident | specialist | of primary care |
| resident | specialist | of internal medicine |
| resident | specialist | of paediatrics |

different answer …
